# ﻿Species richness under a vertebral stripe: integrative taxonomy uncovers three additional species of *Pholidobolus* lizards (Sauria, Squamata, Gymnophthalmidae) from the north-western Colombian Andes

**DOI:** 10.3897/zookeys.1141.94774

**Published:** 2023-01-19

**Authors:** Adolfo Amézquita, Luis A. Mazariegos-H, Santiago Cañaveral, Catalina Orejuela, Leidy Alejandra Barragán-Contreras, Juan M. Daza

**Affiliations:** 1 Laboratory of Biodiversity and Cloud Forests Conservation, Bioconservancy.org, Jardín, Colombia Laboratory of Biodiversity and Cloud Forests Conservation, Bioconservancy.org Jardín Colombia; 2 Department of Biological Sciences, Universidad de los Andes, Bogotá, Colombia Universidad de los Andes Bogotá Colombia; 3 Grupo Herpetológico de Antioquia, Universidad de Antioquia, Medellín, Colombia Universidad de Antioquia Medellín Colombia

**Keywords:** Cryptic species, elevation, leaf-litter lizards, phenotypic space, tropical Andes

## Abstract

The systematic study of biodiversity underlies appropriate inference in most other fields of biological research, yet it remains hampered by disagreements on both theoretical and empirical issues such as the species concept and the operational diagnosis of a species. Both become particularly challenging in those lineages where morphological traits are evolutionarily constrained by their adaptive value. For instance, cryptic organisms often conserve or converge in their external appearance, which hinders the recognition of species boundaries. An integrative approach has been adopted to study microgeographic variation in the leaf-litter lizard *Pholidobolusvertebralis* and test three predictions derived from the evolutionary species concept. Molecular data provided unambiguous evidence of divergence among the three recovered new clades and a common evolutionary history for each of them. The broadly sympatric clades were indeed diagnosable from externally visible traits, such as head scales, adult size, and sexually dimorphic ventral colouration. Also, they barely overlapped on the phenotypic space that summarised 39 morphometric and meristic traits. These clades are described as three species and an available name is suggested for a recovered fourth clade. The geographic distribution of the new and proximate species suggests a role for elevation on evolutionary divergence; it also raises interesting questions on the speciation pattern of an otherwise underestimated cryptic lineage.

## ﻿Introduction

The external appearance of many animal species is known to reduce the probability of being detected against natural backgrounds, which arguably impedes eventual predation at its earliest stage: detection (Krebs and Davies 1993). Therefore, natural selection has promoted the evolution of roughly similar appearance in some species that share microhabitats, no matter the phylogenetic distance that separates them. Because microhabitats vary at very small spatial scales, natural selection has also driven the persistence of multiple colouration patterns within a single species, which presumably reduces the learning rate and the formation of a prey search image by potential predators (Ruxton et al. 2008). Both across-species resemblance and within-species variation may play against the interest of systematics and taxonomy. Many lineages are often hidden under a similar appearance and a single name, until alternative sources of evidence and thorough analyses reveal deep evolutionary divisions among them (Sites-Jr. and Marshall 2004). The combination of molecular, morphological, and distributional data has been instrumental to decipher the limits and the relationships within species complexes, once believed to represent a single evolutionary lineage (Padial et al. 2010).

Lizards in many species bear a mid-dorsal vertebral stripe or line. It presumably contributes to conceal them against the leaf litter or vegetation, by mimicking the petiole and midrib of dead leaves, and thereby breaking their body silhouettes to the eyes of potential predators (Ruxton et al. 2004). The occurrence of vertebral stripes across phylogenetically distant species of lizards (e.g., in the neotropical families Gymnophthalmidae, Dactyloidae, Sphaerodactylidae, Teiidae, and Tropiduridae; Avila-Pires 1995) arguably reflects evolutionary convergence. Their existence across closely related species may instead reflect evolutionary conservatism of a valuable survival trait. To the untrained eye, it may also conceal the existence of well differentiated lineages and complex evolutionary histories. Both convergence and conservatism explain the low value of cryptic colouration in taxonomic studies, which claim instead for multiple sources of evidence to avoid overlooking diversity hidden under a single taxonomic entity.

The neotropical lizards in the genus *Pholidobolus* (Gymnophthalmidae) are distributed throughout the mid to high elevation Andes, from northern Peru and throughout Ecuador up to Colombia (Hurtado-Gómez et al. 2018; Parra et al. 2020). Most intrageneric diversity has been acknowledged in the southern part of its distribution with the recent description of a new species in Peru (Venegas et al. 2016) and four in Ecuador (Parra et al. 2020). Instead, Colombian populations of *Pholidobolus* have been frequently and deliberately assigned to *P.vertebralis* (Hurtado-Gómez et al. 2018), a species bearing a hallmark mid-dorsal vertebral stripe, which indeed inspired its epithet (O’Shaughnessy 1879). The species was known to present both within- and among-localities morphometric variation, which caused some taxonomic instability (Hernández-Ruz and Bernal-González 2011; Doan and Cusi 2014; Venegas et al. 2016). The availability of new specimens and molecular data led to the description of a new species of *Pholidobolus* in high-elevation Paramos of Colombia, *P.paramuno* (Hurtado-Gómez et al. 2018). We became aware of additional geographic variation in body size and ventral colouration in the north-western Colombian Andes, which led us to operationally recognise three morphs occurring as close as 1.5–3.2 km (pairwise map distances) from each other. We aimed at testing whether the available and newly collected evidence were compatible with the existence of multiple lineages, where each (1) had accumulated enough molecular differences as to infer a common evolutionary history, (2) could be diagnosed based on morphometric traits, and (3) exhibited a geographic distribution that convincingly allowed reproductive exchange among individuals of the same morph, but not from different morphs. Those lineages fulfilling at least the first two conditions (Sites-Jr. and Marshall 2004; de Queiroz 2005; de Queiroz 2007), are herein recognised and described as new species.

## ﻿Materials and methods

### ﻿Study area

To conduct phylogenetic and morphometric analyses, we collected specimens along the northern half of the Colombian Western Andes (Cordillera Occidental), and in the northern extreme of the Colombian Central Andes (Cordillera Central), at elevations between 1400–3100 m (Suppl. material 1). The area encloses hills with remnants of cloud forest and small patches of elfin forests; the foothills are mainly covered by a matrix of regenerating forests, cattle pastures, and crops. Around half of the individuals were collected within a small range where three out of the four studied forms occur: the 3500-ha Mesenia-Paramillo Nature Reserve (**MPNR**), ~ 14 km south-west of the municipality of Jardín (Antioquia). According to a weather station at the visitor’s centre, at 2170-m elevation, precipitation is typically bimodal with peaks in April and October, and total annual values exceeding 5000 mm; the average temperature is 15 °C, with maximum daily fluctuations of 10–23 °C.

### ﻿Phylogenetic analyses

To build a phylogenetic hypothesis including the studied lizards, we extracted DNA from liver and muscle samples. Lizards were previously euthanised with an overdose of lidocaine and most of them were photographed, in dorsal and ventral views, against a standard white background. They were afterwards fixed in 10% formalin, and finally stored in 70% ethanol. We extracted DNA using either the Qiagen DNeasy or the GeneJET genomic DNA purification kits and following the standard manufacturer’s protocols for tissue samples. We assembled a molecular matrix including four genomic regions: three mitochondrial and one nuclear region. A fragment of the 12S ribosomal gene was amplified using the primers 12Sa and 12Sb (Kocher et al. 1989), the 16S ribosomal gene using the primers 16SCL and 16SDH (Santos et al. 2003), the protein-coding gene NADH dehydrogenase subunit 4 using the primers ND4 and Leu (Arévalo et al. 1994), and the nuclear protein-coding genes oocyte maturation factor MOS using the primers G73 and G74 (Saint et al. 1998).

We aligned each region using MAFFT under default parameters (Katoh and Standley 2013), and simultaneously estimated the best partition scheme and the model evolution using ModelFinder (Kalyaanamoorthy et al. 2017). We inferred a maximum likelihood tree and nodal support using the ultrafast bootstrap method on 5000 pseudoreplicates as implemented in IQTREE (Hoang et al. 2018; Minh et al. 2020). Lastly, to graphically represent the genetic differentiation among the focal lineages of this study, i.e., those with adjacent, parapatric or overlapping distributions in the north-western Colombian Andes, we estimated uncorrected genetic distances between individuals using MEGA (Kumar et al. 2016). We added *P.vertebralis* to this group, because it is the species to which many of the addressed specimens had been formerly assigned (Hurtado-Gómez et al. 2018).

### ﻿Meristic and morphometric data

We examined and measured 101 individuals. To record meristic traits, we followed definitions for *Pholidobolus* lizards originally proposed by Montanucci (1973), adopted for other gymnophthalmids by Harris (1994) and Kizirian (1996), and later implemented for new species of *Pholidobolus* (Venegas et al. 2016; Hurtado-Gómez et al. 2018): number of pre-frontal scales (**PF**), supraoculars (**SPO**), superciliaries (**SC**), lower palpebrals (**LP**), suboculars (**SO**), postoculars (**PO**), temporal (**TE**), supralabials (**SL**), infralabials (**IL**), pregulars (**PG**), gulars (**GU**), and collar scales (**CL**); we also counted the number of dorsal transverse rows of scales (**DT**), dorsal longitudinal rows (**DL**), transversal ventral rows (**TV**), and the number of scale rows around mid-body (**SAM**). In addition, we took the following morphometric measurements on 94 adult individuals using a digital calliper and to the nearest 0.01 mm: adult snout-vent length (SVL), head length (**HL**), head width (**HW**), head height (**HH**), jaw length (**JL**), snout length (**SL**), length of the longest finger (**LLF**), length of the longest toe (**LLT**), pelvis width (**PW**), and tail width (**TW**). Tail length was measured but excluded from multivariate analyses, because many individuals showed signs of a regenerated tail.

To test whether the recovered lineages could be differentiated from the combined set of meristic and morphometric traits, we conducted discriminant analysis coupled to principal component analysis, DAPC (Jombart et al. 2010). In brief, DAPC aims at discriminating groups of individuals and predicting group membership by using a linear combination of intercorrelated descriptors previously summarised by principal components analysis (PCA). Although PCA alone is often used to graphically depict among-groups variation in multiple traits, this technique is unsupervised, i.e., it is focused on trait covariation yet ignores the group identity of individuals, which increases the risk of overlooking differences among the compared groups (Jombart 2008; Jombart et al. 2010). To conduct DAPC, we used as output variable the identity of the four clades recovered in the phylogenetic analysis; as input variables, we used all meristic and morphometric traits. To account for among-traits differences in variance, the variables were centred to the mean = 0 and scaled, i.e., divided by their estimated standard deviation. To estimate the minimum number of principal components that best summarised morphology and predicted clade membership, we used cross-validation with 90% of the data as training set and the remaining 10% as validation set. At each level of PCA retention, we used 10000 replicates to estimate the mean successful assignment of lizards to the correct clade, and the concomitant value of root mean square error (RMSE). We then selected the number of PCs that maximised the former and minimised the latter, or a lower number when the contribution of additional PCs was considered negligible, i.e., < 1% of successful assignments. We finally ran the definitive DAPC with the selected number of principal components and picked the lowest number of discriminant functions that eventually led to a classification success > 90%. All analyses in this section were conducted on the R package ADEGENET (Jombart and Ahmed 2011).

Male ventral colouration differed at first sight among the clades. To visualise and validate photographic comparisons of the colour hues, we took ventral pictures of both males and females, and adjusted them for white balance using the plugin Auto White Balance Correction Master on the software FIJI (Schindelin et al. 2015); the original macro was written by Vytas Bindokas (Univ. of Chicago). To quantitatively illustrate colour differences among the three males selected as new species’ holotypes, we additionally conducted image segmentation analyses on the R package RECOLORIZE (Weller et al. 2021), grouping slightly varying hues into a lower number of categories with the method kmeans, and depicting the corresponding result on the sRGB colour space.

The hemipenes of two of the three defined holotypes were extracted following Savage (1997), filled with petroleum jelly, and stained with Alizarin 75%. For description of external morphology, we used the terminology in Dowling and Savage (1960), modified for lizards by Savage (1997). The hemipenes were later preserved in 70% alcohol and deposited together with the holotypes at the MHUA: Museo de Herpetología Universidad de Antioquia, Medellín, Colombia. The hemipenis of the third holotype specimen was revised in situ. To facilitate comparisons with all the closely related species of *Pholidobolus*, we gathered similar data from previously published material (LaMarca and García-Pérez 1990; Hernández-Ruz 2005; Hernández-Ruz and Bernal-González 2011; Doan and Cusi 2014; Hurtado-Gómez et al. 2018; Parra et al. 2020).

## ﻿Results

### ﻿Phylogenetic relationships

**Figure 1.**
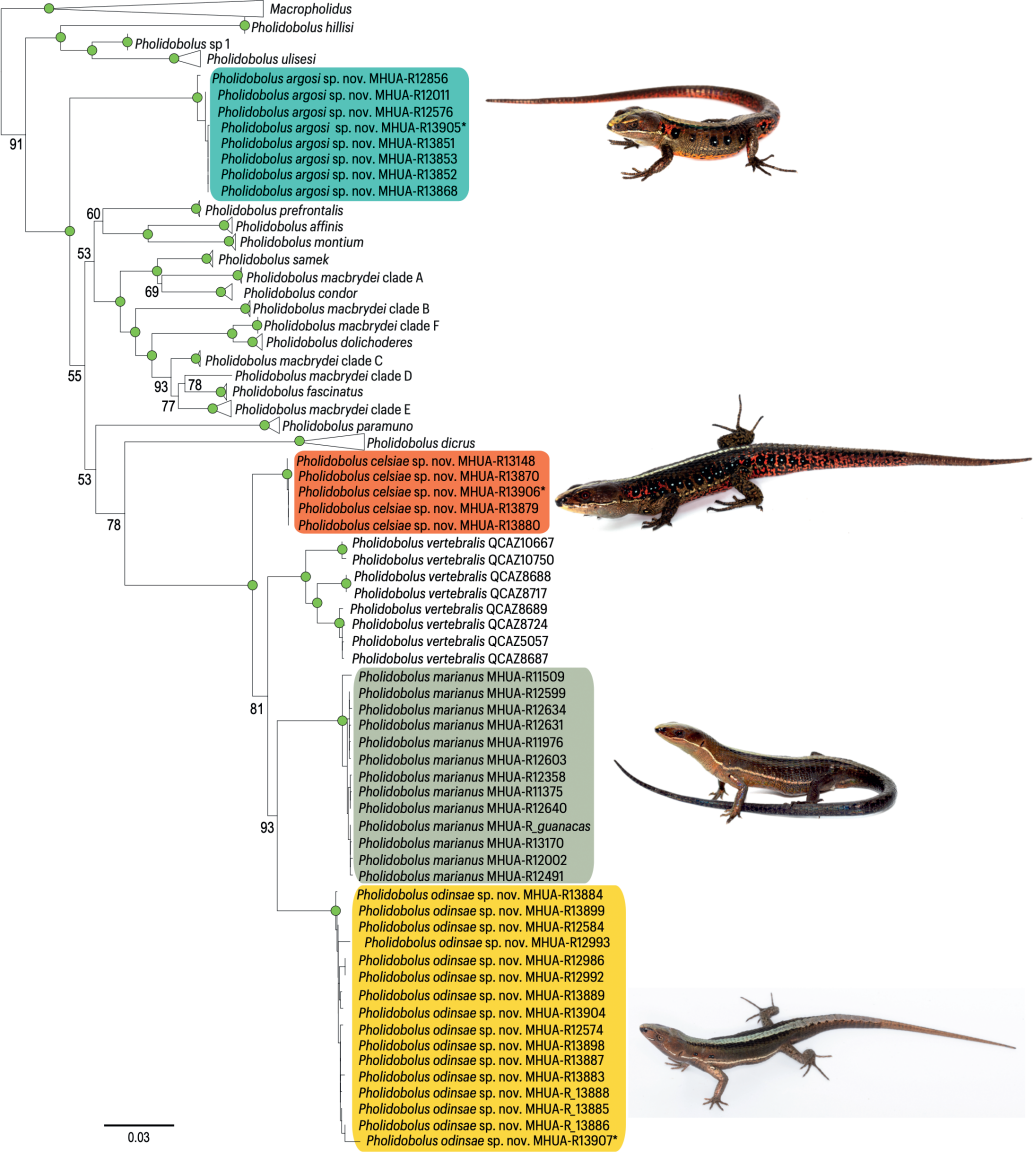
Molecular phylogenetic hypothesis. Recovered relationship between species of the leaf-litter lizards in the genus *Pholidobolus*, including *P.vertebralis* near its type locality in Ecuador. The three recovered clades from the Colombian Western Andes are outlined in blue (clade A), orange (clade B) and yellow (clade D). A fourth clade of the Colombian Central Andes is outlined in green (clade C). Green dots indicate nodal support of at least 95%. See Methods for further details on the phylogenetic analysis and Suppl. material 2 for the detailed tree with individuals as terminal nodes.

The reconstructed phylogenetic hypothesis (Fig. 1) grouped the lizards we sampled into four clades, each with nodal support above 95%. None of them was nested within the specimens currently assigned to *P.vertebralis* in Ecuador (Parra et al. 2020), where the type locality has been situated (Uzzell 1973). Two of them (clades C and D in Fig. 1) were sister clades with a nodal support of 93%, yet indeed distributed in different mountain chains: the Western and Central Colombian Andes. Another one (clade B in Fig. 1) was sister to the clade including the former two and the Ecuadorian specimens currently assigned to *P.vertebralis*, and the fourth (clade A in Fig. 1) was sister to a clade including all the previously mentioned plus one Colombian (*P.paramuno*) and nine Ecuadorian species of *Pholidobolus*, including the polyphyletic *P.macbrydei* (Parra et al. 2020).

**Figure 2.**
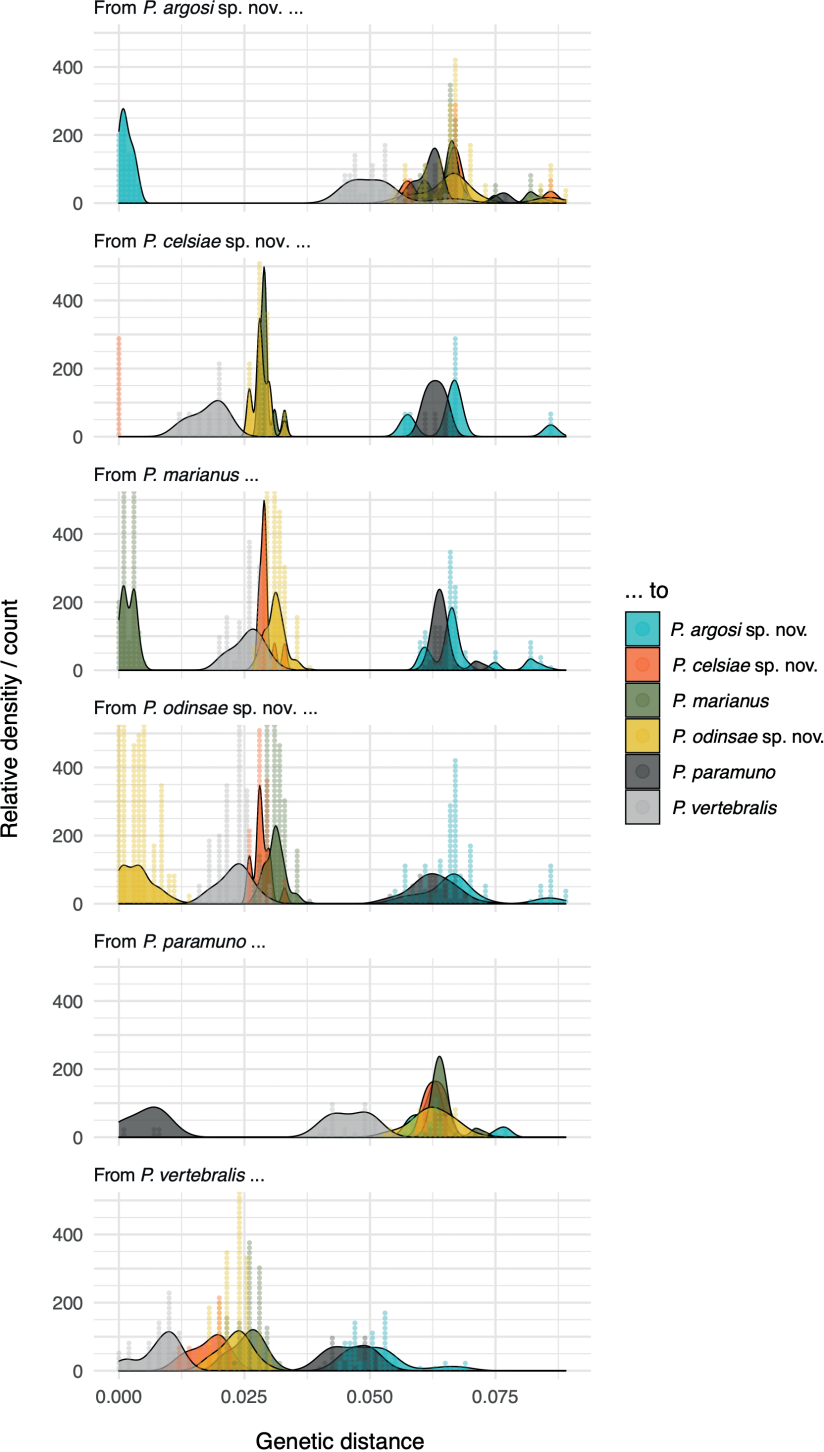
Within and among clades genetic distances. Distribution of uncorrected 16S genetic distances among individuals of leaf-litter lizards in the genus *Pholidobolus*. We include four species of the northern Colombian Andes, and *P.vertebralis* near its type locality in Ecuador. Pairwise distances among individuals of the same clade are indicated with the same colour; pairwise distances among individuals from different clades are represented by different colours. Dots denote each of the calculated distances, whose distribution is summarised by Kernel density smooths.

The distribution of pairwise genetic distances was clearly discontinuous among the lineages with adjacent, parapatric or overlapping distribution in the north-western Colombian Andes: much shorter among individuals of the same recovered clade than among individuals of different clades (Fig. 2). Indeed, the within-clade distribution did not overlap with the among-clades distribution of distances. The sole exception were the Ecuadorian individuals currently assigned to *P.vertebralis*, to which many of the addressed specimens had been formerly assigned. They exhibited the largest within-clade variation. Among the three sympatric lineages of the Colombian Western Andes, which we erect as new species hereafter, genetic distances ranged 2.5–6.3% (Table 1, Fig. 2).

**Table 1.** Genetic distances. Average uncorrected genetic distances among the species of *Pholidobolus* with adjacent, parapatric or overlapping distributions in the north-western Colombian Andes. The species to which some of these individuals had been formerly added, *P.vertebralis* is added for comparison.

Species	*Pa*	*Pc*	*Pm*	*Po*	*Pp*	*Pv*
*P.argosi* sp. nov.		0.066	0.066	0.065	0.060	0.047
*P.celsiae* sp. nov.	0.066		0.029	0.027	0.060	0.014
*P.marianus*	0.066	0.029		0.028	0.061	0.021
*P.odinsae* sp. nov.	0.065	0.027	0.028		0.058	0.017
*P.paramuno*	0.060	0.060	0.061	0.058		0.040
*P.vertebralis*	0.047	0.014	0.021	0.017	0.040	

### ﻿Uniqueness and variation

**Figure 3.**
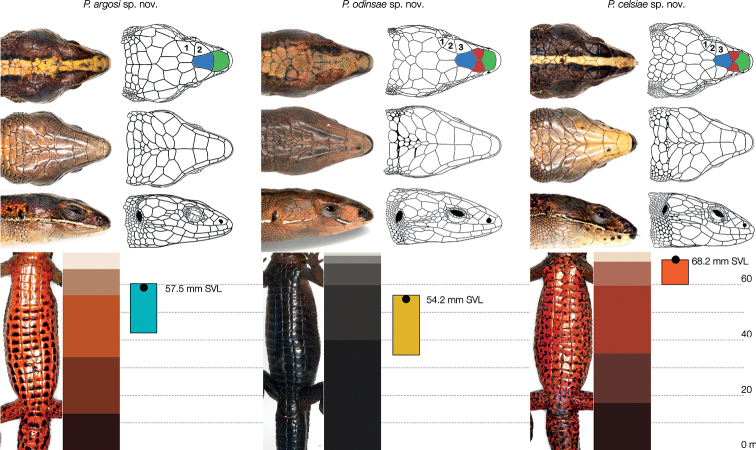
Key morphological features of the three new species on the holotypes. External morphological traits allowing unambiguous diagnosis of the three north-western Colombian clades of *Pholidobolus* lizards recovered in our phylogenetic analysis. *Pholidobolusargosi* sp. nov. lacks one supraocular (numbered) and the prefrontal (filled in red) scales, both of which are present in the other two species. Males of *P.odinsae* sp. nov. exhibit predominantly black and dark grey ventral colouration, which is red with black markings in males of the two other species, as further shown by the image segmentation analyses: the stacked areas denote the proportion of pixels with each summarised hue. Lastly, *P.celsiae* sp. nov. reaches larger adult body size than the other two species, as evidenced by blue (clade A in Fig. 1), orange (B), and yellow (D) bars, which denote range of variation, and the black points, that represent the actual body size of the shown holotypes.

The three clades of the Colombian Western Andes (Clades A / B / D in Fig. 1) were unique and diagnosable regarding the combination of four externally visible traits (Fig. 3): the existence of a prefrontal scale (absent / present / present), the number of supraocular scales (2 / 3 / 3–4), the dominant hue of male ventral colouration (pink to orange / orange / grey to black), and the range of variation in male body (snout to vent) length; 42.6–57.9 (*n* = 15 males), 60.0–68.2 (*n* = 9), and 35.4–54.7 mm (*n* = 22).

**Figure 4.**
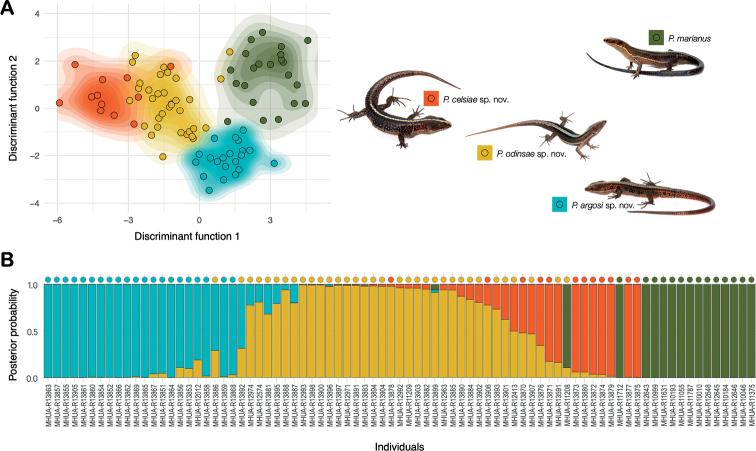
Discriminant and classification analysis based on lizard morphology. Classification analysis of lizards in four species of *Pholidobolus* based on the whole set of meristic and morphometric traits **A** distribution of individuals (dots) of the recovered phylogenetic clades (colours) in the two-dimensional phenotypic space created by the discriminant analysis of principal components (DAPC) summarising all traits **B** actual (dot colour) and predicted (bar colour) membership of each lizard (museum identity) to the recovered clades; predicted membership is estimated from the DAPC and represents the probability of assignment of each lizard to one or more lineages (colours). See Methods for further detail on the underlying statistical analyses.

Regarding the whole set of morphometric and meristic traits, the first cross-validation analysis indicated that seven principal components summarised enough variation as to attempt phenotypic discrimination and classification of individuals. The existence of near discrete clades was further supported by the scatter of individuals throughout the phenotypic space created by the two first discriminant axes (Fig. 4A). We recovered four consistent clusters with bare overlap among them, three corresponding to the Western Andean clades and one from the Central Andean clade; only four out of 81 individuals were plotted in the wrong cluster. Indeed, the resulting DAPC correctly assigned 91% of the revised specimens to the corresponding phylogenetic clade (Fig. 4B). According to the dimensionless loading coefficients of DAPC, which vary between 0–1, the largest contributions of standardised traits to the discriminating task were from the number of dorsal longitudinal scale rows (0.30), the number of ventral transverse scale rows (0.18), and the number of lower palpebrals (0.18).

**Figure 5.**
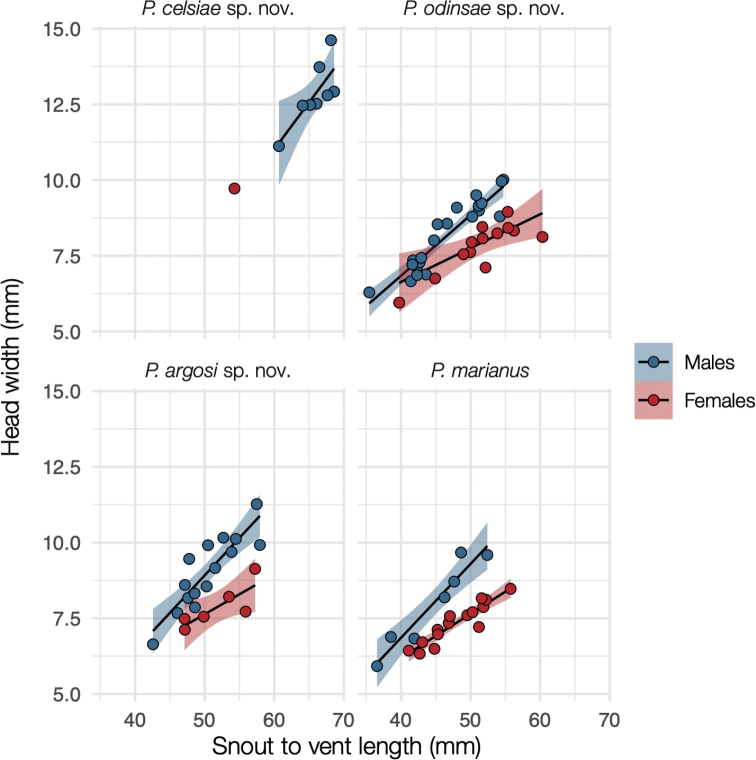
Sexual dimorphism in the lizard head width. Relationship between head width and body size across four species of *Pholidobolus* lizards. Males exhibit disproportionally wider heads compared to females. Only one female was available for *P.celsiae* sp. nov. Lines denote linear regression, and the coloured shadows indicate the 95% confidence interval of the line slope. Non-overlapping blue and red shadows represent significant differences in the slope of the head width to body size relationship, between males and females.

**Figure 6.**
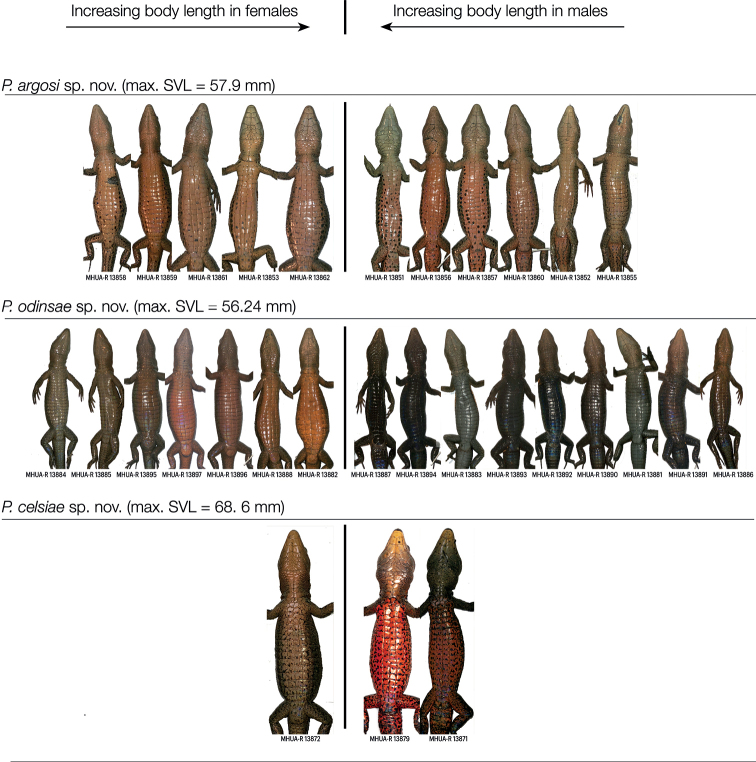
Variation in lizard ventral colouration. Among-species, among-sexes, and among-individuals variation in ventral colouration of three lizard species in the genus *Pholidobolus*. Pictures were taken immediately after euthanisation to reflect colour in life. To depict eventual covariation with body size, individuals are sorted from the smallest (extremes) to the largest (middle) one.

Regarding sexual dimorphism, males had wider heads than expected from their body size, though the pattern could not be corroborated in one clade due to the capture of a single female (Fig. 5). There was also sexual dichromatism, with females being generally pink to pale orange, or cream in the two clades (A and D) we could check (Fig. 6). In contrast, males of the Clade A (Fig. 1) were pink to pale orange, with more black marks than females; in the Clade D (Fig. 1) they were black to medium grey; and in the Clade B (Fig. 1), they were bright orange with black blotches on the scale edges (Fig. 6). The smallest, and presumably youngest, individuals of all clades were usually grey to cream, and lacked the black patterning (Fig. 6).

### ﻿Geographic distribution

**Figure 7.**
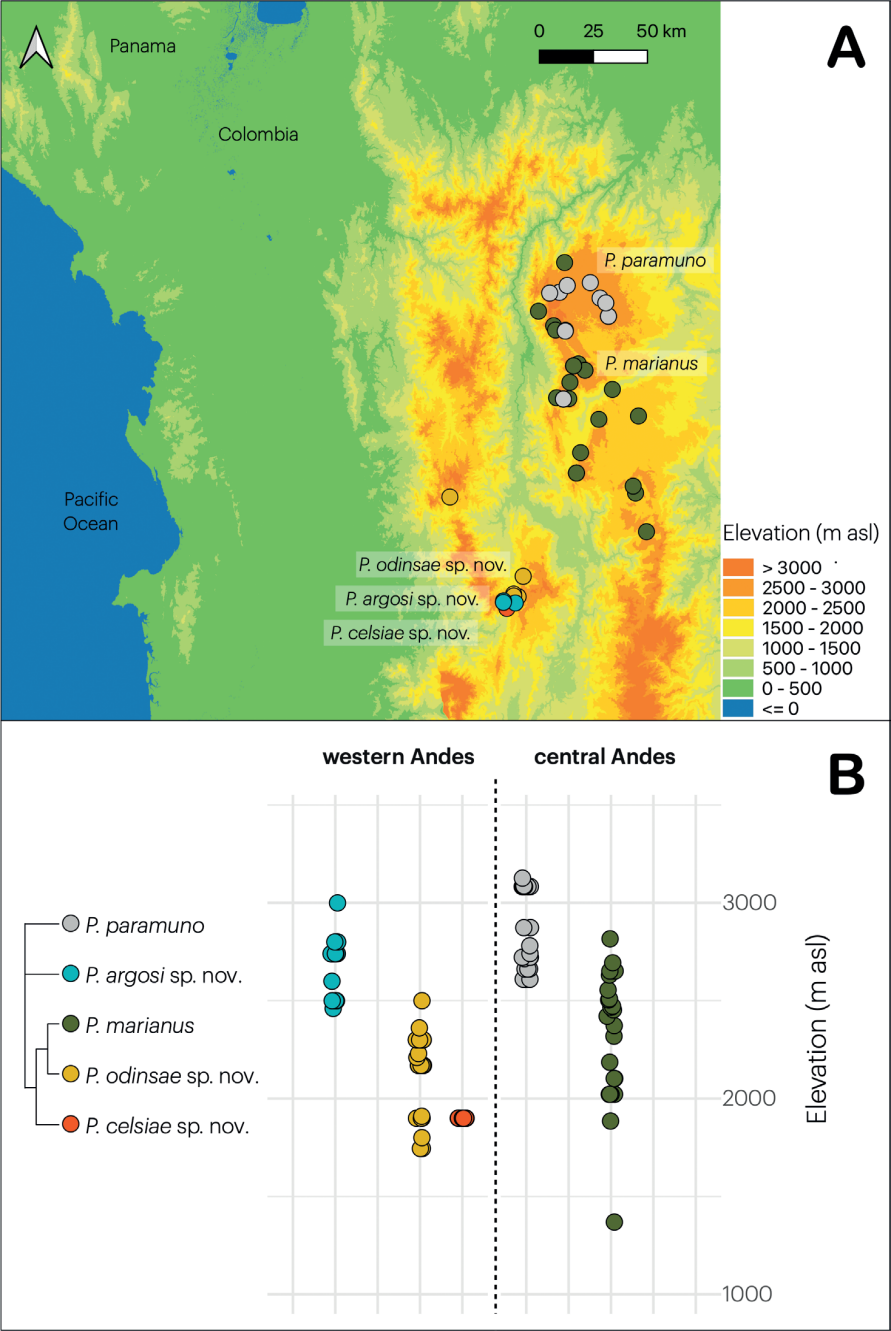
Geographic and altitudinal distribution. Geographic (**A**) and altitudinal (**B**) distribution of five species of *Pholidobolus* lizards across the north of the Western and Central Colombian Andes. The cladogram summarises the recovered phylogenetic relationship among them (Fig. 1). Each dot represents an individual deposited in the MHUA-R collection at the Universidad de Antioquia.

During this and parallel studies, all but one individual were captured at altitudes above 1500 m and up to 3100 m. They were thus absent in the lower elevation valley of the Cauca River, which separates the Western and Central Andean chains of Colombia. Among-clades differences in distribution were thus best described in terms of Andean chain and elevation. One clade (clade C in Fig. 1) was found exclusively on the Central Andes (Fig. 7A), where they exhibit some degree of altitudinal segregation with the recently described *P.paramuno* (Hurtado-Gómez et al. 2018): between 1900–2800 for the former and between 2600–3100 m elevation for the latter (Fig. 7B). Among the Western Andean clades (Fig. 7A), there was some degree of altitudinal segregation as well (Fig. 7B). One (clade D in Fig. 1) occurred in the north-eastern slopes, between 1700–2500 m; the second (clade A) in the hilltops, 2400–3000 m a.s.l.; and the third one (clade B) was found at 1900 m elevation in the south-western slopes. Remarkably, the two clades occurring at elevations below 2500 m were found on opposite slopes and thereby basins of the Western Andes (Fig. 7A). The slope-elevation segregation was more evident in the MPNR, where they occur within a radius of 3.5 km without evidence of syntopy, despite our intensive search in the area.

### ﻿Systematics

Based on the collected samples and evidence, we recognise the existence of four phylogenetically independent lineages. A name is available for the specimens from the northern Central Andes (Cordillera Central) in Colombia (Clade C in Fig. 1). The species *Prionodactylus* [*Pholidobolus*] *marianus* (Ruthven 1921) was described from San Pedro (Antioquia, Colombia) based on specimens collected by the late Brother Nicéforo Maria on March 25, 1921. The religious community Hermanos de La Salle owns a retreat house at the municipality of San Pedro de Los Milagros (Antioquia, Colombia, 75°33.60'W, 6°27.60'N), a small town less than 20 km north to the city of Medellín. San Pedro (its short name) is a well-known collection locality particularly associated to the work by Brother N. María and Brother M.A. Serna (Donegan et al. 2009), both pivotal contributors and curators of the Natural History Museum at the Universidad de La Salle. The reported altitude where the *P.marianus* holotype was collected (2560 m elevation [Uzzell 1973]) can be found less than 200 m away from the retreat house. Because the specimens we collected at San Pedro (MHUA-R12643, MHUA-R12645, MHUA-R12646, MHUA-R12648) are nested in our analyses within the green clade (Figs 1, 2, 4, 5, 7), we propose to resurrect the name *Pholidobolusmarianus* for all specimens in this clade (Figs 1, 4, 7).

The specimens of the Colombian Western Andes (Cordillera Occidental) were grouped into three diagnosable (Fig. 3), morphologically distinctive (Fig. 4), genetically discontinuous (Fig. 2), and phylogenetically independent (Fig. 1) clades. None of them was nested in our analyses within the Ecuadorian specimens assigned to *P.vertebralis*; moreover, they were found near 650 km north of its type locality (Uzzell 1973). Therefore, we erect three new species for these lineages and provide below formal descriptions of them. To facilitate actual and future comparisons with other *Pholidobolus* of the northern Colombian Andes, we followed Hurtado-Gómez et al. (2018) regarding terminology and descriptions.

#### ﻿ Pholidobolus argosi sp. nov.

Taxon classificationAnimaliaSquamataGymnophthalmidae

8C5D42D0-BCD6-5699-BBBC-45F8CD11C476

https://zoobank.org/63913FC3-DE51-4941-8D35-08A8BF38FE67

Figs 1 , 3 , 6

##### Type material.

***Holotype*.** (Figs 1, 3; Table 2). Adult male. Field original label: “AA_7058.” Museum ID: MHUA-R13905. Type locality in Colombia, Antioquia: municipality of Andes, 5°29.92'N, 75°54.27'W, 2500 m elevation, Mesenia-Paramillo Nature Reserve, in secondary forest, amidst the leaf litter, 7 October 2020. Collected by Ubiel Rendón and Luis A. Mazariegos-H.

**Table 2.** Type series. Identity, sex and geographic location of type specimens of the three new species of *Pholidobolus* lizards described here.

Species	Voucher	Type	Field code	Sex	Locality	Elev (m)	GPS coordinates
*P.argosi*	MHUAR13905	holotype	AA7058	male	MPNR	2500	5°29.92'N, 75°54.27'W
*P.argosi*	MHUAR12011	paratype		juvenile	Santa Rita	2730	5°34.75'N, 75°57.68'W
*P.argosi*	MHUAR12012	paratype		male	Santa Rita	2730	5°34.75'N, 75°57.68'W
*P.argosi*	MHUAR13851	paratype	AA7010	male	MPNR	2500	5°29.92'N, 75°54.27'W
*P.argosi*	MHUAR13852	paratype	AA7014	male	MPNR	2740	5°29.54'N, 75°54.31'W
*P.argosi*	MHUAR13853	paratype	AA7017	female	MPNR	2740	5°29.54'N, 75°54.31'W
*P.argosi*	MHUAR13854	paratype	AA7039	male	MPNR	2500	5°29.92'N, 75°54.27'W
*P.argosi*	MHUAR13855	paratype	AA7048	male	MPNR	2740	5°29.54'N, 75°54.31'W
*P.argosi*	MHUAR13856	paratype	AA7049	male	MPNR	2740	5°29.54'N, 75°54.31'W
*P.argosi*	MHUAR13857	paratype	AA7050	male	MPNR	2740	5°29.54'N, 75°54.31'W
*P.argosi*	MHUAR13858	paratype	AA7051	female	MPNR	2740	5°29.54'N, 75°54.31'W
*P.argosi*	MHUAR13859	paratype	AA7052	female	MPNR	2740	5°29.54'N, 75°54.31'W
*P.argosi*	MHUAR13860	paratype	AA7053	male	MPNR	2740	5°29.54'N, 75°54.31'W
*P.argosi*	MHUAR13861	paratype	AA7054	female	MPNR	2740	5°29.54'N, 75°54.31'W
*P.argosi*	MHUAR13862	paratype	AA7055	female	MPNR	2740	5°29.54'N, 75°54.31'W
*P.argosi*	MHUAR13863	paratype	AA7059	male	MPNR	2500	5°29.92'N, 75°54.27'W
*P.argosi*	MHUAR13864	paratype	AA7066	male	MPNR	2840	5°28.76'N, 75°54.37'W
*P.argosi*	MHUAR13865	paratype	AA7067	male	MPNR	2840	5°28.76'N, 75°54.37'W
*P.argosi*	MHUAR13866	paratype	AA7068	male	MPNR	2840	5°28.76'N, 75°54.37'W
*P.argosi*	MHUAR13867	paratype	AA7179	female	MPNR	2500	5°29.92'N, 75°54.27'W
*P.argosi*	MHUAR13868	paratype	AA7180	male	MPNR	2490	5°29.39'N, 75°51.35'W
*P.argosi*	MHUAR13869	paratype	AA7181	male	MPNR	2840	5°28.76'N, 75°54.37'W
*P.celsiae*	MHUAR13906	holotype	AA7061	male	MPNR	1900	5°28.01'N, 75°53.44'W
*P.celsiae*	MHUAR13148	paratype		juvenile	Mampay	1720	5°21.51'N, 75°52.91'W
*P.celsiae*	MHUAR13520	paratype		male	La Suiza	1830	4°43.93'N, 75°35.09'W
*P.celsiae*	MHUAR13870	paratype	AA7002	male	MPNR	1900	5°28.01'N, 75°53.44'W
*P.celsiae*	MHUAR13871	paratype	AA7056	male	MPNR	1900	5°28.01'N, 75°53.44'W
*P.celsiae*	MHUAR13872	paratype	AA7057	female	MPNR	1900	5°28.01'N, 75°53.44'W
*P.celsiae*	MHUAR13873	paratype	AA7069	male	MPNR	1900	5°28.01'N, 75°53.44'W
*P.celsiae*	MHUAR13874	paratype	AA7070	male	MPNR	1900	5°28.01'N, 75°53.44'W
*P.celsiae*	MHUAR13875	paratype	AA7071	male	MPNR	1900	5°28.01'N, 75°53.44'W
*P.celsiae*	MHUAR13876	paratype	AA7072	male	MPNR	1900	5°28.01'N, 75°53.44'W
*P.celsiae*	MHUAR13877	paratype	AA7073	male	MPNR	1900	5°28.01'N, 75°53.44'W
*P.celsiae*	MHUAR13878	paratype	AA7074	female	MPNR	1900	5°28.01'N, 75°53.44'W
*P.celsiae*	MHUAR13879	paratype	AA7161	male	MPNR	1900	5°28.01'N, 75°53.44'W
*P.celsiae*	MHUAR13880	paratype	AA7172	male	MPNR	1900	5°28.01'N, 75°53.44'W
*P.odinsae*	MHUAR13907	holotype	AA7090	male	MPNR	2180	5°29.76'N, 75°53.35'W
*P.odinsae*	MHUAR12574	paratype		male	Santa Rita	2150	5°35.52'N, 75°57.15'W
*P.odinsae*	MHUAR12584	paratype		juvenile	Quebradona	2240	5°45.38'N, 75°43.37'W
*P.odinsae*	MHUAR12986	paratype		juvenile	La Isla	1730	5°51.50'N, 76°9.73'W
*P.odinsae*	MHUAR13883	paratype	AA7009	male	MPNR	1920	5°31.62'N, 75°51.75'W
*P.odinsae*	MHUAR13884	paratype	AA7011	female	MPNR	1920	5°31.62'N, 75°51.75'W
*P.odinsae*	MHUAR13885	paratype	AA7012	female	MPNR	1920	5°31.62'N, 75°51.75'W
*P.odinsae*	MHUAR13886	paratype	AA7013	male	MPNR	2300	5°31.13'N, 75°51.74'W
*P.odinsae*	MHUAR13887	paratype	AA7015	male	MPNR	1920	5°31.62'N, 75°51.75'W
*P.odinsae*	MHUAR13888	paratype	AA7016	female	MPNR	2210	5°29.62'N, 75°53.40'W
*P.odinsae*	MHUAR13889	paratype	AA7019	juvenile	MPNR	2300	5°31.13'N, 75°51.74'W
*P.odinsae*	MHUAR13898	paratype	AA7182	female	MPNR	2310	5°29.46'N, 75°53.33'W
*P.odinsae*	MHUAR13899	paratype	AA7183	female	MPNR	2310	5°29.46'N, 75°53.33'W
*P.odinsae*	MHUAR13904	paratype	AA7188	male	MPNR	2230	5°30.96'N, 75°50.63'W

***Paratypes*.** Fourteen males, six females, and one juvenile. Table 2 shows field codes, localities, elevation, and geographic coordinates. Eighteen specimens were collected in Colombia, Antioquia: municipality of Andes, Mesenia-Paramillo Nature Reserve (MPNR), and one in Colombia, Caldas: municipality of Riosucio, MPNR, years 2018, 2019, and 2020. Collected by Ubiel Rendón, Luis A. Mazariegos, Jorge Jaramillo, and Osman López. The two other specimens from Colombia, Antioquia: Andes, Santa Rita, year 2009. Collected by Cornelio Bota.

##### Diagnosis.

The species can be diagnosed combining the following characters: (1) two supraocular scales; (2) prefrontal scales absent; (3) 9–17 temporal scales; (4) dorsal scales keeled; (5) 28–32 transverse rows of dorsal scales; (6) 20–22 transverse rows of ventral scales; (7) 26–35 scales around mid-body; (8) 1–2 (usually 1) rows of lateral scales; (9) lateral and medial ventral scales equal in size; (10) 0–5 femoral pores; (11) no sexual dimorphism in number of femoral pores; (12) labial scales pale, often crossed dorsally by a longitudinal white stripe bordered with black; (13) ventral head colouration paler towards the anterior end; (14) cream or white vertebral stripe bordered by two black stripes, originating on the rostral scale, completely covering the dorsal region of the head and the vertebral region of the body, reaching only the anterior portion of the tail, with maximum width of four scales on the body; (15) lateral colour brown, orange towards the shoulders and anterior part of the tail, with some ocelli, usually less than seven between limbs insertions, each white in centre and surrounded by black scales, with a longitudinal white line in the head, pale and discontinuous towards the body; (16) venter pink to pale orange, with few black markings in females; vivid orange with much more and much larger black markings in adult males; (17) subcylindrical and bilobed hemipenial body with 6–8 and 7–9 rows of spinulated flounces in the lateral columns of the sulcate and asulcate sides, respectively; (18) lateral columns of spinulated flounces connecting in the proximal region of the asulcate side.

##### Comparisons.

*Pholidobolusvertebralis* differs from *P.argosi* sp. nov. (character states in parenthesis) in having the lateral ventral scales smaller than the medial ventrals (lateral and medial ventral scales equal in size). The other species from the north-western and central Colombian Andes (Fig. 7) differ from *P.argosi* sp. nov. in having prefrontal scales (absent), and 3–4 supraocular scales (2). In addition, males in *P.paramuno* are ventrally reddish brown and in *P.odinsae* sp. nov. are black to grey (pink to pale orange). Lastly, males of *P.celsiae* sp. nov. are larger in size (Table 3), between 60.7–68.6 mm of snout–vent length (42.6–57.9 mm).

**Table 3.** Meristic and morphometric traits. Summary of meristic and morphometric (in mm) traits in adult lizards of the four clades of *Pholidobolus* studied here. Mean ± sd (min – max).

Trait	*P.argosi* sp. nov. (*n* = 21)	*P.celsiae* sp. nov. (*n* = 11)	*P.marianus* comb. nov. (*n* = 24)	*P.odinsae* sp. nov. (*n* = 35)
Prefrontals	0 ± 0 (0–0)	2.0 ± 0.0 (2–2)	2.0 ± 0.0 (2–2)	2.0 ± 0.0 (2–2)
Supraoculars	2.0 ± 0.0 (2–2)	3.0 ± 0.0 (3–3)	3.4 ± 0.5 (3–4)	3.0 ± 0.2 (3–4)
Superciliaries	3.9 ± 0.4 (3–5)	3.9 ± 0.3 (3–4)	3.7 ± 0.6 (3–5)	4.0 ± 0.2 (3–5)
Lower palpebrals	3.9 ± 0.7 (2–5)	4.4 ± 0.7 (3–5)	5.4 ± 1.1 (4–7)	3.9 ± 0.8 (2–5)
Suboculars	4.1 ± 0.6 (3–5)	4.9 ± 0.9 (4–6)	3.9 ± 0.8 (3–6)	5.2 ± 1.1 (3–7)
Postoculars	2.0 ± 0.0 (2–2)	2.1 ± 0.3 (2–3)	2.9 ± 0.6 (2–4)	2.2 ± 0.5 (2–4)
Temporal	12.5 ± 1.8 (9–17)	21.8 ± 4.8 (14–28)	13.8 ± 2.7 (10–19)	19.9 ± 3.3 (11–26)
Supralabials	6.9 ± 0.4 (6–8)	7.2 ± 0.4 (7–8)	6.9 ± 0.5 (6–8)	7.1 ± 0.4 (7–8)
Infralabials	5.6 ± 0.6 (5–7)	4.6 ± 0.7 (4–6)	4.9 ± 0.7 (3–6)	4.8 ± 0.7 (4–6)
Pregulars	2.1 ± 0.2 (2–3)	2.4 ± 0.5 (2–3)	3.8 ± 0.9 (2–6)	2.3 ± 0.6 (2–4)
Gulars	7.1 ± 0.4 (6–8)	7.6 ± 0.5 (7–8)	7.9 ± 1.3 (4–10)	8.1 ± 0.6 (7–9)
Collar scales	10.3 ± 1.5 (7–13)	11.2 ± 1.6 (9–14)	10.3 ± 1.7 (6–13)	10.9 ± 2.0 (6–17)
Dorsal transverse	30.1 ± 1.0 (28–32)	29.5 ± 0.9 (28–31)	30.3 ± 1.2 (28–32)	29.9 ± 1.0 (28–32)
Dorsal longitudinal	18.8 ± 1.2 (17–21)	24.9 ± 1.0 (23–26)	22.3 ± 1.9 (19–26)	22.5 ± 2.0 (20–26)
Around mid-body	31.3 ± 2.3 (26–35)	39.6 ± 1.5 (37–43)	35.9 ± 2.6 (30–42)	38.4 ± 3.4 (31–45)
Transversal ventral	21.1 ± 0.7 (20–22)	19.6 ± 1.0 (18–21)	21.2 ± 1.2 (19–24)	19.8 ± 1.2 (17–23)
Head width	8.70 ± 1.20 (6.6–11.3)	11.89 ± 1.20 (7.4–14.6)	7.53 ± 0.98 (5.9–9.7)	8.02 ± 1.02 (6.0–10.0)
Head length	11.77 ± 1.79 (9.1–15.0)	15.05 ± 2.46 (10.4–17.9)	10.66 ± 1.56 (7.6–14.5)	11.24 ± 1.39 (8.6–15.0)
Head height	6.13 ± 0.87 (5.0–7.7)	8.44 ± 1.51 (5.2–9.7)	5.32 ± 0.71 (3.9–7.1)	5.41 ± 0.63 (4.2–6.7)
Jaw length	9.51 ± 1.42 (7.2–13.2)	12.77 ± 1.56 (9.4–14.5)	10.15 ± 1.88 (7.1–14.1)	9.67 ± 1.50 (7.4–13.2)
Longest finger	5.25 ± 0.58 (4.1–6.4)	5.80 ± 0.79 (4.7–6.8)	4.75 ± 0.47 (4.0–5.6)	4.72 ± 0.63 (3.9–6.3)
Pelvis width	6.57 ± 0.70 (4.8–7.6)	8.60 ± 1.01 (6.2–9.6)	6.57 ± 0.91 (4.2–7.9)	6.67 ± 0.97 (4.9–8.9)
Longest toe	8.13 ± 0.77 (6.8–10.2)	9.42 ± 0.90 (7.4–10.2)	7.19 ± 0.65 (5.7–8.2)	7.19 ± 1.06 (5.3–10.8)
Tail width	5.50 ± 0.59 (4.3–6.6)	7.61 ± 1.42 (5.1–9.5)	4.97 ± 0.60 (4.1–6.1)	5.69 ± 0.97 (3.9–8.1)
Snout-vent length	50.85 ± 4.23 (42.6–57.9)	62.38 ± 7.19 (45.0–68.6)	46.97 ± 4.81 (35.6–55.8)	48.36 ± 5.71 (35.4–60.3)

##### Description of the holotype.

Adult male; snout-vent length 57.5 mm; tail length 111.0 mm; other body measurements can be found in Table 4. Head scales smooth, juxtaposed, glossy, with small pits organised mainly around their margins. Rostral single, hexagonal, wider than high, dorsally in broad contact with the internasal and laterally in contact with the first supralabial and the nasal. Frontonasal single, wider than long, hexagonal, in contact with the nasal, loreal and the frontal one. Prefrontal scales absent. Frontal single, pentagonal, longer than wide, wider anteriorly, in contact with the frontonasal. Frontoparietals two, pentagonal, longer than wide, narrower anteriorly, contacting the first two supraoculars laterally, and the parietal and interparietals posteriorly. Supraoculars two, wider than longer and increasing in size antero-posteriorly, contacting the superciliaries laterally and the parietal and postocular posteriorly. Interparietal single, hexagonal, longer than wide, narrower than the parietals and contacting laterally the parietals and posteriorly the postparietals. Parietals two, pentagonal, wider than long, slightly shorter and wider than the interparietal, contacting the temporals laterally and the postparietals posteriorly. Postparietals in two rows, two in the anterior row and four in the posterior row. Nasal single, rhomboidal, wider than high, contacting the first and second supralabials, the loreal and frenocular. Loreal single, quadrangular, over the frenocular, in contact with first superciliary dorsally. Frenocular single, triangular, in contact with the first infraocular and the second and third supralabials. Superciliaries three, the anteriormost noticeable larger than the others, contacting the uppermost preocular. Suboculars four contacting supralabials three to five. Postoculars two, the dorsal one larger than the ventral one. Temporals 13, contacting supralabials five to seven. Supralabials seven and infralabials six. Mental single, pentagonal, wider than long, contacting the first infralabial and postmental. Postmental single, pentagonal, contacting the first three infralabials and the anterior genials. Genials in three pairs, the anterior one quadrangular, the posteriors pairs pentagonals and larger than the anterior one, contacting infralabials three, three and four, and five respectively. Pregulars two. Gular scales seven, wider than long, in two longitudinal rows; collar scales ten, decreasing in size laterally. Dorsal scales longer than wide, hexagonal, keeled, imbricate, arranged in 31 transverse rows. Longitudinal rows of dorsal scales 17, the first two rows in each side weakly keeled and rounded. Lateral row scales at mid-body one, smooth, at least half the size of adjacent scales. Scales around mid-body 31. Longitudinal rows of ventrals eight, quadrangular. Transverse rows of ventrals 18. Cloacal plates in two rows of two scales each, the anterior one quadrangular, the posterior row rounded, larger than the anterior one. Tail scales arranged in 80 rings, hexagonal and keeled dorsally, quadrangular and smooth ventrally.

**Table 4.** Holotypes. Sex, body measurements (in mm), and voucher identity of the holotypes of the new lizard species describe herein.

Trait	*P.argosi* sp. nov.	*P.celsiae* sp. nov.	*P.odinsae* sp. nov.
Sex	male	male	male
Snout-vent length	57.5	68.2	54.2
Head length	14.9	16.6	11.2
Head width	11.3	14.6	8.8
Head height	7.6	9.7	6.3
Jaw length	10.5	12.8	10.9
Length of the longest finger	6.4	6.1	4.9
Length of the longest toe	8.8	10.0	7.4
Pelvis width	7.6	9.6	6.3
Tail length	111.0	79.0	50.0
Tail width	6.4	9.5	5.0

Limbs pentadactyl with clawed fingers. Dorsal brachial and antebrachial scales lanceolate to polygonal, longer than wide, imbricate and smooth. Ventral brachial and antebrachial scales lanceolate to polygonal, almost as long as wide, juxtaposed, much smaller than the dorsal ones. Dorsal hand scales hexagonal, wider but shorter than the dorsal antebrachial scales. Finger length formula IV > III > II > V > I. Supradigital scales quadrangular, imbricate and wider than long. Palmar scales polygonal, juxtaposed, and small. Subdigital lamellae domelike with a quadrangular base, and often divided longitudinally, with six on finger I, 10 on II, 12 on III, 15 on IV, and 10 on V. Thigh scales on the dorsal, anterior and ventral surfaces lanceolate to rhomboidal, longer than wide, those in the dorsal surface smooth and the others smooth and imbricate. Thigh scales on the posterior surface of the legs rounded, smooth, juxtaposed and much smaller than those of the anterior and dorsal surfaces. Five femoral pores per leg; preanal pores absent. Anterior and ventral crus scales polygonal and smooth. Lateral and posterior crus scales rounded, small and subimbricate. Toe length formula IV > III > V > II > I. Supradigital scales quadrangular, imbricate and longer than wide. Plantar scales polygonal, juxtaposed and small. Subdigital lamellae domelike with a quadrangular base, and often divided longitudinally, with six on Toe I, 10 on II, 16 on III, 20 on IV, and 11 on V.

##### Colouration.

In life, dorsally brown, bisected by a mid-dorsal (i.e. vertebral) cream, pale brown, or white stripe, extending from the head to the base of the tail; vertebral stripe bordered with darker, usually black, stripes; on the head, the pale stripe extends from the first supralabial to the shoulder dorsally reaching the rostral scale, and laterally bordering the supraocular and parietal scales; sides of neck, flanks, and limbs predominantly brown, usually with less than ten white ocelli, bordered by a black stripe; white or cream lateral line from the supralabials to the shoulder; cream and interrupted lateral stripe, running between the insertions of fore and hind limbs, not extending towards the tail; scattered red scales, more common and grouped above the shoulder and along the lateral surface of the tail; throat pink to cream; chest, belly and base of the tail pink to pale orange, often with black blotches, apparently more common in adult males (Figs 1, 3, 6). In preservative, brown surfaces become paler, the dorsal and lateral stripes become white, and the red surfaces on the flanks, chest, belly and tail fade to white or very pale pink.

##### Etymology.

The species epithet is dedicated to the Grupo Argos Foundation, for their commitment to sustainable development, and their voluntary actions directed to education and environmental restoration. Through its program “Sembrando Futuro”, they promote the conservation and recovery of water resources, depleted gallery forests, mangroves, and the habitat of the spectacled bear, an umbrella species for the conservation of entire Andean ecosystems.

##### Distribution, ecology, and conservation.

The species is currently known from the hilltops of the western Andes, near the municipalities of Andes and Caramanta, within the department of Antioquia. Most specimens were seen amongst the leaf litter of elfin forests; some were collected on secondary forests at the edge of cloud forests. The observed specimens appeared clearly heliothermic: within minutes after the sun appeared, they came out of the leaf litter, remained exposed, and extended their ribs increasing the dorsal surface available for sunlight capture. Under sunny conditions, several individuals could be seen at once in at least two of the spots from where the species is known. Its distribution seems thus to be very patchy, known presently from fewer than five locations and in any case less than 500 km^2^ (Fig. 7). The cloud and elfin forests are severely fragmented in the area, and remain mainly as small patches on hilltops, which are preserved to protect water sources for crops downhill. Therefore, until new information is collected, we suggest listing the new species as Endangered EN B1ab(iii), B2ac(iii), under the IUCN criteria (IUCN 2012). Many individuals showed signs of a regenerated tail.

#### ﻿ Pholidobolus celsiae sp. nov.

Taxon classificationAnimaliaSquamataGymnophthalmidae

AB37609B-D693-5ED0-838E-2E7907FEA8D8

https://zoobank.org/A75418D8-7BB3-4764-848B-4ADBD2E12D47

##### Type material.

***Holotype*.** Adult male, with genitalia in a separate microvial. Original label: AA_7061. Museum ID: MHUA-R13906. Type locality in Colombia, Risaralda: Municipality of Mistrató, 5°28.01'N, 75°53.44'W, secondary forest, under rocks, 7 October 2020. Collected by Ubiel Rendón and Luis A. Mazariegos-H.

***Paratypes*.** Eleven males, two females, and one juvenile. Table 2 shows field codes, localities, elevation, and geographic coordinates. Twelve specimens were collected in Colombia, Risaralda: Mistrató, Mesenia-Paramillo Nature Reserve (MPNR), May 2018, June 2019, and October 2020. Collected by Ubiel Rendón, Luis A. Mazariegos-H., Jorge Jaramillo, and Osman López. One from Colombia: Risaralda, Municipality of Mistrató, Mampay village. Collected by Juan P. Hurtado. The other from Colombia, Risaralda: Municipality of Pereira, vereda La Suiza, Santuario de Fauna y Flora Otún Quimbaya. Collected by Melisa Galeano.

##### Diagnosis.

The species can be diagnosed combining the following characters: (1) three supraocular scales; (2) prefrontal scales present; (3) 14–28 temporal scales; (4) dorsal scales keeled; (5) 28–32 transverse rows of dorsal scales; (6) 18–21 transverse rows of ventral scales; (7) 36–44 scales around mid-body; (8) 1–3 rows of lateral scales; (9) lateral and medial ventral scales equal in size; (10) 5–6 femoral pores; (11) no sexual dimorphism in number of femoral pores; (12) labial scales pale, often with black markings; (13) ventral head colouration homogeneous in females; with irregular orange or black markings, and paler towards the anterior half in males; (14) white to cream vertebral stripe bordered by two black stripes, originating on the rostral scale, completely covering the dorsal region of the head and the vertebral region of the body, reaching only the anterior portion of the tail, with maximum width of two scales on the body; (15) lateral colour pattern brown and dark orange to red, with numerous ocelli, usually more than seven between the limbs insertions, white in centre and surrounded by black scales, with a longitudinal pale line laterally, continuous and white in the head, pale and discontinuous towards the body; (16) venter pink to pale orange, or brown, with darker marking towards the edge of scales in females; vivid orange to red, with scattered black markings towards the edge of scales in males; (17) subcylindrical and bilobed hemipenial body with 4–5 and 7–9 rows of spinulated flounces in the lateral columns of the sulcate and asulcate sides, respectively; (18) lateral columns of spinulated flounces connecting in the distal region of the asulcate side.

##### Comparisons.

*Pholidobolusvertebralis* differs from *P.celsiae* sp. nov. (character states in parenthesis) in having the lateral ventral scales smaller than the medial ventrals (lateral and medial ventral scales equal in size). The other species from the north-western and central Colombian Andes (Fig. 7) differ from *P.celsiae* sp. nov. in exhibiting smaller adult body size in males (Table 2), between 35.4–54.7 mm in *P.odinsae* sp. nov., and 42.6–57.9 mm in *P.argosi* sp. nov. (60.7–68.6 mm). In addition, males of *P.argosi* sp. nov. lack prefrontal scales (present) and have two supraocular scales (3–4). Lastly, males of *P.odinsae* sp. nov. exhibit black to grey and males of *P.paramuno* reddish brown ventral coloration (orange).

##### Description of the holotype.

Adult male; snout-vent length 68.2 mm; tail length 79.0 mm; other body measurements in Table 4. Head scales smooth, juxtaposed, glossy, with small pits organised mainly around their margins. Rostral single, hexagonal, wider than high, dorsally in broad contact with the internasal and laterally in contact with the first supralabial and the nasal. Frontonasal single, wider than long, pentagonal, in contact with the nasal, loreal and prefrontals. Prefrontals two, wider laterally and narrower medially, in wide contact with the first superciliary, the frontal and the anterior supraocular. Frontal single, hexagonal, longer than wide, wider anteriorly, in contact with the prefrontals, the first supraocular and the frontoparietals. Frontoparietals two, pentagonal, longer than wide, narrower anteriorly, contacting the first two supraoculars laterally, and the parietal and interparietals posteriorly. Supraoculars three, the anteriormost nearly as wide as long and the other two wider than long, decreasing in size antero-posteriorly, contacting the superciliaries laterally and the parietal and postocular posteriorly. Interparietal single, heptagonal, longer than wide, narrower than the parietals and contacting laterally the parietals and posteriorly the postparietals. Parietals two, hexagonal, wider than long, slightly shorter and wider than the interparietal, contacting the temporals laterally and the postparietals posteriorly. Postparietals in two rows, three in the anterior row and four in the posterior row. Nasal single, wider than high, contacting the first and second supralabials, the loreal and frenocular. Loreal single, quadrangular, over the frenocular, in contact with first superciliary dorsally. Frenocular single, triangular, in contact with the first infraocular and the second and third supralabials. Superciliaries four, the anteriormost noticeable larger than the others, contacting the uppermost preocular. Suboculars five contacting supralabials three to five. Postoculars two, ventral larger than dorsal. Temporals 17 contacting supralabials five to seven. Supralabials seven and infralabials five. Mental single, pentagonal, wider than long, contacting the first infralabial and postmental. Postmental single, pentagonal, contacting the first two infralabials and the anterior genials. Genials in three pairs, the anterior one quadrangular and the posterior two pentagonal. The anterior two in contact medially and the posterior one separated by postgenials; contacting infralabials two, three, and four. Pregulars two. Gular scales seven, wider than long, in two longitudinal rows; collar scales 13 decreasing in size laterally. Dorsal scales longer than wide, hexagonal, keeled, imbricate, arranged in 29 transverse rows. Longitudinal rows of dorsal scales 23, the first two rows in each side weakly keeled and rounded. Lateral row scales at mid-body one, smooth, at least half the size of adjacent scales. Scales around mid-body 39. Longitudinal rows of ventrals six, quadrangular. Transverse rows of ventrals 20. Cloacal plates in two rows of two quadrangular scales each, the posterior row larger than the anterior one, in contact with two small scales laterally. Tail scales arranged in 54 rings, hexagonal and keeled dorsally, quadrangular and smooth ventrally.

Limbs pentadactyl with clawed fingers. Dorsal brachial and antebrachial scales lanceolate to polygonal, almost as long as wide, imbricate and smooth. Ventral brachial and antebrachial scales lanceolate to polygonal, almost as long as wide, juxtaposed, much smaller than the dorsal ones. Dorsal hand scales hexagonal, wider but shorter than the dorsal antebrachial scales. Finger length formula IV = III > II > V > I. Supradigital scales quadrangular and imbricate. Palmar scales polygonal, juxtaposed and small. Subdigital lamellae domelike with a quadrangular base, and often divided longitudinally, with four on finger I, 8 on II, 12 on III, 13 on IV, and 7 on V. Thigh scales on the dorsal, anterior and ventral surfaces lanceolate to rhomboidal, longer than wide, those in the dorsal surface keeled and the others smooth and imbricate. Thigh scales on the posterior surface of the legs rounded, smooth, juxtaposed and much smaller than those of the anterior and dorsal surfaces. Five femoral pores per leg; preanal pores absent. Anterior and ventral crus scales polygonal and keeled. Lateral and posterior crus scales rounded, small and subimbricate. Toe length formula IV > III > II > IV > I. Supradigital scales quadrangular, imbricate and longer than wide. Plantar scales polygonal, juxtaposed and small. Subdigital lamellae domelike with a quadrangular base, and often divided longitudinally, with four on Toe I, 8 on II, 13 on III, 15 on IV, and 9 on V. Thigh scales on the dorsal, anterior and ventral surfaces lanceolate to rhomboidal, longer than wide, those in the dorsal surface keeled and the others smooth and imbricate. Thigh scales on the posterior surface of the legs rounded, smooth, juxtaposed and much smaller than those of the anterior and dorsal surfaces. Five femoral pores per leg; preanal pores absent. Anterior and ventral crus scales polygonal and keeled. Lateral and posterior crus scales rounded, small and subimbricate. Toe length formula IV > III > V > II > I. Supradigital scales quadrangular, imbricate and longer than wide. Plantar scales polygonal, juxtaposed and small. Subdigital lamellae domelike with a quadrangular base, and often divided longitudinally, with seven on I, nine on II, 13 on III, 18 on IV, and 10 on V.

##### Colouration.

In life, dorsally dark brown, bisected by a mid-dorsal (i.e. vertebral) cream, or white stripe, extending from the head to the base of the tail; vertebral stripe bordered with darker, usually black, stripes; on the head, the pale stripe extends from the first supralabial to the shoulder dorsally reaching the rostral scale, and laterally not in contact with the supraocular and parietal scales; sides of neck, flanks, and limbs predominantly brown; neck, flanks and tail base usually with more than 10 white ocelli, bordered by a black stripe; white or cream lateral line from the supralabials to the shoulder; cream and interrupted lateral stripe, running between the insertions of fore and hind limbs, not extending towards the tail; many red scales, more common in males and grouped above the shoulder and along the lateral surface of the tail; throat cream to pale brown in males, paler towards the anterior extreme; throat pink in females; chest, belly and base of the tail cream to pink in females, but orange in males, often with black blotches, apparently more common in adult males (Figs 1, 3, 6). In preservative, brown surfaces become paler, the dorsal and lateral stripes become white, and the red surfaces on the flanks, chest, belly and tail fade to white or very pale pink.

##### Etymology.

The species epithet is dedicated to the Celsia Foundation, for their voluntary contribution to the restoration of cloud and dry forests in the tropical Andes, through their reforestation program Reverde-C, which already planted more than one million trees. In addition, their program for children education in rural areas, already benefited more than 16000 students in terms of school infrastructure, teacher training, and further logistic support during the Covid pandemic. We believe their commitment contributes to the well-being and education of direct neighbours and thereby stakeholders of Colombian protected nature.

##### Distribution, ecology, and conservation.

The specimens were mostly collected in open areas with secondary vegetation, at the edge of a cloud forest. Groups of up to nine eggs were found together with adult individuals under a rock, suggesting communal nesting. Also, the observed specimens appeared clearly heliothermic: within minutes after the sun appeared, they came out of their refuges, remained exposed, and extended their ribs increasing the dorsal surface available for sun basking. The species is currently known from three localities, two of them within protected areas: the Mesenia-Paramillo Nature Reserve, and the Santuario de Flora y Fauna (SFF) Otún-Quimbaya. Further explorations are needed to ascertain the species distribution. In the meantime, we suggest listing the new species as Endangered EN B1ab(iii), B2ac(iii), under the IUCN criteria (IUCN 2012). Many individuals showed signs of a regenerated tail.

#### ﻿ Pholidobolus odinsae sp. nov.

Taxon classificationAnimaliaSquamataGymnophthalmidae

4E599355-79EC-5471-A1FA-8D6843068DD4

https://zoobank.org/9C94C382-240C-4E33-9C91-24C1363959FF

##### Type material.

***Holotype*.** (Figs 1, 3) Adult male, with genitalia in a separate microvial. Original field label: AA_7090. Museum ID: MHUA-R13907. Type locality in Colombia, Antioquia: Municipality of Jardín, Mesenia-Paramillo Nature Reserve, 5°29.76'N, 75°53.35'W, visitor centre, among pastures, 14 November 2020. Collected by Ubiel Rendón and Luis A. Mazariegos-H.

***Paratypes*.** Six males, five females, and three juveniles. Table 2 shows field codes, localities, elevation, and geographic coordinates. Eleven specimens were collected in Colombia, Antioquia: Municipality of Jardín, Mesenia-Paramillo Nature Reserve (MPNR), between June 2018 and June 2020. Collected by Osman López, Ubiel Rendón, Jorge Jaramillo, and Luis A. Mazariegos. One from Colombia, Antioquia: Municipality of Andes, vereda Santa Rita, El Chaquiro. One from Colombia, Antioquia: Municipality of Jericó, vereda Quebradona, Finca La Aurora. The other from Colombia, Chocó: Municipality of Carmen de Atrato, vereda La Isla, Finca Gualandai.

##### Diagnosis.

The species can be diagnosed combining the following characters: (1) 3–4 (usually 3) supraocular scales; (2) prefrontal scales present; (3) 11–28 temporal scales; (4) dorsal scales keeled; (5) 28–32 transverse rows of dorsal scales; (6) 17–23 transverse rows of ventral scales; (7) 31–45 scales around mid-body; (8) 3–5 rows of lateral scales; (9) lateral and medial ventral scales equal in size; (10) 0–2 femoral pores; (11) no sexual dimorphism in number of femoral pores; (12) labial scales similar in colour to other head scales, crossed by a curved pale lip line, best described as two oblique white lines converging in the eye; (13) ventral head colouration homogeneous; (14) cream or white vertebral stripe bordered by two black stripes, originating on the rostral scale, completely covering the dorsal region of the head and the vertebral region of the body, reaching only the anterior portion of the tail, with maximum width of four scales on the body; (15) lateral colour pattern brown, with a complete longitudinal line laterally, white and continuous from the posteroventral edge of the ear until the insertion of the hind limbs; with very few ocelli usually above the insertion of the forelimbs and absent between the limbs insertions, small; ocelli white in centre and surrounded by black scales and, beyond that, sometimes a few reddish scales; (16) venter strongly dimorphic in colouration between the sexes, uniformly pink to pale orange in females, sometimes with very few black speckles but no markings; usually glossy black and sometimes medium grey in males; (17) hemipenial body with 7–8 and 11–12 rows of spinulated flounces in the lateral columns of the sulcate and asulcate sides, respectively; (18) lateral columns of spinulated flounces connecting in the medial region of the asulcate side.

##### Comparisons.

*Pholidobolusvertebralis* differs from *P.odinsae* sp. nov. (character states in parenthesis) in having the lateral ventral scales smaller than the medial ventrals (lateral and medial ventral scales equal in size). The other species from the north-western and central Colombian Andes (Fig. 7) differ from *P.odinsae* sp. nov. in ventral colouration of males: reddish brown in *P.paramuno*, and pink to orange in *P.argosi* sp. nov. and *P.celsiae* sp. nov. (black to grey). In addition, males of *P.argosi* sp. nov. lack prefrontal scales (present) and have two supraocular scales (3–4). Lastly, males of *P.celsiae* sp. nov. are larger in size (Table 3), between 60.7–68.6 mm (35.4–54.7 mm).

##### Description of the holotype.

Adult male; snout-vent length 54.2 mm; tail length 50.0 mm; other body measurements in Table 4. Head scales smooth, juxtaposed, glossy, with small pits organized mainly around their margins. Rostral single, hexagonal, wider than high, dorsally in broad contact with the internasal and laterally in contact with the first supralabial and the nasal. Frontonasal single, wider than long, pentagonal, in contact with the nasal, loreal and prefrontals. Prefrontals two, wider laterally and narrower medially, in contact, touching the frontonasal, the frontal, the anterior supraocular, and the loreal. Frontal single, hexagonal, longer than wide, wider anteriorly, in contact with the prefrontals, the first supraocular and the frontoparietals. Frontoparietals pentagonal, longer than wide, narrower anteriorly, contacting one to three supraoculars laterally, and the parietal and interparietals posteriorly. Supraoculars four, the anterior most nearly as wide as long and the other two wider than long, decreasing in size antero-posteriorly, contacting the superciliaries laterally and the parietal and uppermost postocular posteriorly. Interparietal single, heptagonal, longer than wide, narrower than the parietals and contacting laterally the parietals and posteriorly the postparietals. Parietals two, hexagonal, wider than long, slightly shorter and wider than the interparietal, contacting the temporals laterally and the postparietals posteriorly. Postparietals in two rows, three in the anterior row and two in the posterior row. Nasal single, rhomboidal, wider than high, contacting the first and second supralabials, the loreal and frenocular. Loreal single, quadrangular, over the frenocular, in contact with first superciliary dorsally. Frenocular single, quadrangular in contact with the first infraocular and the second and third supralabials. Superciliaries three, the anteriormost noticeable larger than the others, contacting the uppermost preocular. Suboculars five, contacting supralabials three to five. Postoculars two, increasing in size antero-posteriorly. Temporals 26, contacting supralabials five to eight. Supralabials eight and infralabials six. Mental single, pentagonal, wider than long, contacting the first infralabial and post-mental. Postmental single, pentagonal, contacting the first two infralabials and the anterior genials. Genials in three pairs, the anterior one quadrangular and the posterior two pentagonal. The anterior two in contact medially and the posterior one separated by postgenials; contacting infralabials two, three, and four. Pregulars two. Gular scales eight, wider than long, in two longitudinal rows; collar scales 17, decreasing in size laterally. Dorsal scales longer than wide, hexagonal, keeled, imbricate, arranged in 30 transverse rows. Longitudinal rows of dorsal scales 24, the first two rows in each side weakly keeled and rounded. Lateral row scales at mid-body one, smooth, at least half the size of adjacent scales. Scales around mid-body 45. Longitudinal rows of ventrals six, quadrangular. Transverse rows of ventrals 20. Cloacal plates in two rows of two quadrangular scales each, the posterior row larger than the anterior one, in contact with two small scales laterally. Tail scales arranged in 62 rings, hexagonal and keeled dorsally, quadrangular and smooth ventrally.

Limbs pentadactyl with clawed fingers. Dorsal brachial and antebrachial scales lanceolate to polygonal, longer than wide, imbricate and smooth. Ventral brachial and antebrachial scales lanceolate to polygonal, almost as long as wide, juxtaposed, much smaller than the dorsal ones. Dorsal hand scales hexagonal, wider but shorter than the dorsal antebrachial scales. Finger length formula IV = III > II > V > I. Supradigital scales quadrangular, imbricate and longer than wide. Palmar scales polygonal, juxtaposed and small. Subdigital lamellae domelike with a quadrangular base, and often divided longitudinally, with six on finger I, 8 on II, 13 on III, 15 on IV, and 7 on V. Thigh scales on the dorsal, anterior and ventral surfaces lanceolate to rhomboidal, longer than wide, those in the dorsal surface smooth and the others smooth and imbricate. Thigh scales on the posterior surface of the legs rounded, smooth, juxtaposed and much smaller than those of the anterior and dorsal surfaces. Two femoral pores per leg; preanal pores absent. Anterior and ventral crus scales polygonal and smooth. Lateral and posterior crus scales rounded, small and subimbricate. Toe length formula IV > III > V > II > I. Supradigital scales quadrangular, imbricate and longer than wide. Plantar scales polygonal, juxtaposed and small. Subdigital lamellae domelike with a quadrangular base, and often divided longitudinally, with five on Toe I, 9 on II, 13 on III, 15 on IV, and 7 on V.

##### Colouration.

In life, dorsally brown or pale brown, bisected by a mid-dorsal (i.e. vertebral) white stripe, extending from the head to the mid tail; vertebral stripe bordered with darker, usually dark brown or black, stripes; on the head, the pale stripe extends from the first supralabial to the shoulder dorsally reaching the rostral scale, and laterally including the frontonasal, prefrontal, frontal, frontoparietal, interparietal, and postparietal scales; sides of neck, flanks, and limbs predominantly brown, usually with less than five, small and white ocelli, bordered by a black stripe, and predominantly on the shoulders; white or cream lateral line from the supralabials, passing through the shoulder and extending continuously up to the insertion of the limbs, but not towards the tail; very few scattered red scales, more common around the shoulder ocelli; throat cream to pink in females, but grey to black in males; chest, belly and base of the tail pink to orange in adult females, but grey to black in males, with bare or no patterning in all cases (Figs 1, 3, 6). In preservative, brown surfaces become paler, the dorsal and lateral stripes become white, the orange chest, belly and tail of females fade to white or very pale pink, and the black chest, belly and tail of males fade into dark grey.

##### Etymology.

The species epithet is dedicated to the company Odinsa, for their decisive involvement in the Cartama Conservation Project, in southwestern Antioquia, aimed at restoring ecosystem services by regenerating the Andean forest along the Quebrada San Antonio basin. Together with other stakeholders, the initiative planted more than 320000 native trees during 2019–2020 alone.

##### Distribution, ecology, and conservation.

The species is currently known from forest edges, and open areas including pastures, crops, and around human buildings. Most specimens were seen and found amongst grass or leaf litter even hundreds of metres away from the nearest forests. They appeared clearly heliothermic: within minutes after the sun appeared, they came out of their refuges, remain exposed, and extended their ribs increasing the dorsal surface available for sun basking. Under sunny conditions, the species seems to be abundant at the known localities. Its distribution seems not to be patchy, and it is known from more than five locations. Although they encompass less than 500 km^2^ (Fig. 7), the actual distribution could arguably exceed this threshold area, given the species adaptability to disturbed habitats. Therefore, we suggest listing the new species as Data Deficient, DD (IUCN 2012), until proper information is collected to evaluate the species conservation status. Many individuals showed signs of a regenerated tail.

## ﻿Discussion

This study provides unambiguous phylogenetic, genetic, morphological, and geographic evidence of independent evolutionary history in four lineages of *Pholidobolus* lizards, formerly assigned to *P.vertebralis*. Based on the available evidence, their evolution would have occurred at mid and high elevation Andes, and led to the origin of several geographically proximate species yet occupying a small area each. The only exception would be *P.vertebralis*, whose status as a single species is thus challenged by at least two lines of evidence.

First, most *Pholidobolus* species occupy small areas. The genus currently comprises 13 species, 11 of which are considered endemic: five are known from a single locality and four others from areas below 7000 km^2^, mostly in Ecuador. To the best of our knowledge, the three new species described herein, and the single species resurrected, exhibit small distribution ranges. In contrast, *P.vertebralis* is the only taxon believed to occur throughout 110000 km^2^ and three countries: Ecuador, Colombia and Venezuela (Doan and Cusi 2014). In Ecuador, a single study including molecular data added four additional species to the genus with small distribution each (Parra et al. 2020); the same is true for the four species addressed in this study, and for a single species in a previous one (Hurtado-Gómez et al. 2018). Altogether, the evidence suggests that the geographic distribution of the putative *P.vertebralis* is extremely atypical, if not an artifact created by the lack of genetic sampling and associated research on the entire distribution range.

Second, the genus distribution appears to exclude low elevation areas. The available evidence supports that most *Pholidobolus* species occur above 1000 m elevation, sometimes on hilltops, but mostly confined to one side of the Andes or the other (Uzzell 1973; Parra et al. 2020). Throughout our study area, two species were indeed found on the hilltops, and two out of three were confined to one of the slopes of the Colombian Central and Western Andes, but no specimens were found from low elevation areas. In contrast, *P.vertebralis* is reported in Colombia throughout the three Andean chains, which are indeed separated by two low elevation areas: the Magdalena and the Cauca river valleys. The species distribution is thus highly atypical, because it includes the opposite sides of likely geographic barriers, the low elevation valleys, and because its area is much larger than for any other species in the genus. Such atypical distribution may instead reflect the existence of several valid species currently assigned to *P.vertebralis*.

If our argument holds, we anticipate that new species will be recognised once enough molecular and morphological data are collected throughout the distribution of *P.vertebralissensu lato.* We also suggest revising published decisions on the validity of species previously regarded as synonyms of *P.vertebralis*. For instance, the main argument to synonymise *Prionodactylus* [*Pholidobolus*] *palmeri* and *P.marianus* was that the holotype “falls within my concept of *P.vertebralis*” (Uzzell 1973). Although the author reported quantitative morphological data, they were not explicitly invoked to support this decision. Likewise, the species *Euspondylus* [*Pholidobolus*] *ampuedae* [*ampuedai*] was synonymised with *P.vertebralis* yet recognising that “I was unable to examine any specimens of *Euspondylus* [*Pholidobolus*] *ampuedae* [*ampuedai*]” (Uzzell 1973), and rooting this decision on the shape of a pale lip line. The species status was later revalidated as *Prionodactylus* [*Pholidobolus*] *ampuedai* by LaMarca and García-Pérez (1990), but again synonymised with *Cercosaura* [*Pholidobolus*] *vertebralis* by Doan and Cusi (2014). This formerly acknowledged species was described from Villa Páez, State of Táchira, Venezuela, near the border with Colombia; it was later thought to be distributed along the Colombian Eastern Andes (Cordillera Oriental), which would render its distribution disjunct with the other Colombian species we address here (Fig. 7A). Behind those decisions was the acknowledgement of large morphological variation within the specimens assigned by then to *P.vertebralis*. Such variation is compatible with a polyphyletic nature of the taxon. To test this hypothesis, a modern analysis of morphological variation and the collection of molecular data are both strictly required.

The findings and arguments exposed here have relevant implications not only for the systematics of the group, but also for the recognition of appropriate conservation units. One of the foremost tasks in conservation is the delineation of biologically meaningful units, which implies the recognition of ecologically discrete and evolutionarily significant lineages. Particularly in the tropics, the task is hampered by the paucity of information on the genotype, phenotype, and geographic distribution of most taxa, which is further aggravated by the contrast between the extremely high level of species richness and the meagre resources devoted to analysing it. During centuries, species recognition was mainly based on detailed written accounts of species external morphology. Now, much more consideration is needed to examine the extent of variation in external morphology that is merely attributable to phylogenetic signal. Morphology is often evolutionarily conserved, due to the adaptive value of the phenotype. Dorsal colouration is cryptic and arguably conserved in *Pholidobolus* species; ventral colouration instead probably plays a crucial role as mate recognition signal and could therefore bear important and overlooked information for species delimitation. The adoption of integrative approaches involving systematics and other branches of biology will probably contribute to untangling true diversity levels, by ascertaining the number and distribution of evolutionarily independent lineages. This information is badly needed to delimit biologically meaningful conservation units, which could allow sound, scientifically based, decisions on conservation actions and priorities.

## Supplementary Material

XML Treatment for Pholidobolus argosi

XML Treatment for Pholidobolus celsiae

XML Treatment for Pholidobolus odinsae

## Supplementary materials

Supplementary material 1 Linked data table for primary biodiversity data Data type occurrences Explanation note Data types and sequenced specimens of *Anadia*, *Macropholidus*, and *Pholidobolus* lizards (Gymnophthalmidae) used to build the phylogenetic hypothesis in this study. https://binary.pensoft.net/file/797363This dataset is made available under the Open Database License (http://opendatacommons.org/licenses/odbl/1.0/). The Open Database License (ODbL) is a license agreement intended to allow users to freely share, modify, and use this Dataset while maintaining this same freedom for others, provided that the original source and author(s) are credited.Adolfo Amézquita, Luis A. Mazariegos-H, Santiago Cañaveral, Catalina Orejuela, Leidy Alejandra Barragán-Contreras, Juan M. Daza

Supplementary material 2 Detailed phylogenetic tree Data type phylogenetic Explanation note Phylogenetic hypothesis on the relationships between studied lizards. Each terminal represents an included individual. https://binary.pensoft.net/file/797364This dataset is made available under the Open Database License (http://opendatacommons.org/licenses/odbl/1.0/). The Open Database License (ODbL) is a license agreement intended to allow users to freely share, modify, and use this Dataset while maintaining this same freedom for others, provided that the original source and author(s) are credited.Adolfo Amézquita, Luis A. Mazariegos-H, Santiago Cañaveral, Catalina Orejuela, Leidy Alejandra Barragán-Contreras, Juan M. Daza
