# Supplementary material for: ﻿Species richness under a vertebral stripe: integrative taxonomy uncovers three additional species of Pholidobolus lizards (Sauria, Squamata, Gymnophthalmidae) from the north-western Colombian Andes
Source: Zookeys. 2023 Jan 19;1141:119–48. doi: 10.3897/zookeys.1141.94774 (PMC10208605; doi:10.3897/zookeys.1141.94774)
Supplement: Supplementary material 1 — Linked data table for primary biodiversity data [file zookeys-1141-119_article-94774__-s001.pdf]

Species	Voucher	Type	Field code	Sex	Department	Municipality	Reserve/ Village	Elevation (m a.s.l.)	Latitude	Longitude
P. argosi sp. nov.	MHUA-R13905	**holotype**	AA_7058	male	Antioquia	Andes	MPNR	2500	5° 29.918' N	75° 54.272' W
P. argosi sp. nov.	MHUA-R12011	paratype		juvenile	Antioquia	Andes	Santa Rita	2730	5° 34.752' N	75° 57.684' W
P. argosi sp. nov.	MHUA-R12012	paratype		male	Antioquia	Andes	Santa Rita	2730	5° 34.752' N	75° 57.684' W
P. argosi sp. nov.	MHUA-R13851	paratype	AA_7010	male	Antioquia	Andes	MPNR	2500	5° 29.918' N	75° 54.272' W
P. argosi sp. nov.	MHUA-R13852	paratype	AA_7014	male	Antioquia	Andes	MPNR	2740	5° 29.536' N	75° 54.307' W
P. argosi sp. nov.	MHUA-R13853	paratype	AA_7017	female	Antioquia	Andes	MPNR	2740	5° 29.536' N	75° 54.307' W
P. argosi sp. nov.	MHUA-R13854	paratype	AA_7039	male	Antioquia	Andes	MPNR	2500	5° 29.918' N	75° 54.272' W
P. argosi sp. nov.	MHUA-R13855	paratype	AA_7048	male	Antioquia	Andes	MPNR	2740	5° 29.536' N	75° 54.307' W
P. argosi sp. nov.	MHUA-R13856	paratype	AA_7049	male	Antioquia	Andes	MPNR	2740	5° 29.536' N	75° 54.307' W
P. argosi sp. nov.	MHUA-R13857	paratype	AA_7050	male	Antioquia	Andes	MPNR	2740	5° 29.536' N	75° 54.307' W
P. argosi sp. nov.	MHUA-R13858	paratype	AA_7051	female	Antioquia	Andes	MPNR	2740	5° 29.536' N	75° 54.307' W
P. argosi sp. nov.	MHUA-R13859	paratype	AA_7052	female	Antioquia	Andes	MPNR	2740	5° 29.536' N	75° 54.307' W
P. argosi sp. nov.	MHUA-R13860	paratype	AA_7053	male	Antioquia	Andes	MPNR	2740	5° 29.536' N	75° 54.307' W
P. argosi sp. nov.	MHUA-R13861	paratype	AA_7054	female	Antioquia	Andes	MPNR	2740	5° 29.536' N	75° 54.307' W
P. argosi sp. nov.	MHUA-R13862	paratype	AA_7055	female	Antioquia	Andes	MPNR	2740	5° 29.536' N	75° 54.307' W
P. argosi sp. nov.	MHUA-R13863	paratype	AA_7059	male	Antioquia	Andes	MPNR	2500	5° 29.918' N	75° 54.272' W
P. argosi sp. nov.	MHUA-R13864	paratype	AA_7066	male	Antioquia	Andes	MPNR	2840	5° 28.725' N	75° 54.367' W
P. argosi sp. nov.	MHUA-R13865	paratype	AA_7067	male	Antioquia	Andes	MPNR	2840	5° 28.725' N	75° 54.367' W
P. argosi sp. nov.	MHUA-R13866	paratype	AA_7068	male	Antioquia	Andes	MPNR	2840	5° 28.725' N	75° 54.367' W
P. argosi sp. nov.	MHUA-R13867	paratype	AA_7179	female	Antioquia	Andes	MPNR	2500	5° 29.918' N	75° 54.272' W
P. argosi sp. nov.	MHUA-R13868	paratype	AA_7180	male	Caldas	Riosucio	MPNR	2490	5° 29.393' N	75° 51.349' W
P. argosi sp. nov.	MHUA-R13869	paratype	AA_7181	male	Antioquia	Andes	MPNR	2840	5° 28.725' N	75° 54.367' W
P. celsiae sp. nov.	MHUA-R13906	**holotype**	AA_7061	male	Risaralda	Mistrató	MPNR	1900	5° 28.014' N	75° 53.443' W
P. celsiae sp. nov.	MHUA-R13148	paratype		juvenile	Risaralda	Mistrató	Mampay	1720	5° 21.509' N	75° 52.909' W
P. celsiae sp. nov.	MHUA-R13520	paratype		male	Risaralda	Pereira	La Suiza	1830	4° 43.931' N	75° 35.092' W
P. celsiae sp. nov.	MHUA-R13870	paratype	AA_7002	male	Risaralda	Mistrató	MPNR	1900	5° 28.014' N	75° 53.443' W
P. celsiae sp. nov.	MHUA-R13871	paratype	AA_7056	male	Risaralda	Mistrató	MPNR	1900	5° 28.014' N	75° 53.443' W
P. celsiae sp. nov.	MHUA-R13872	paratype	AA_7057	female	Risaralda	Mistrató	MPNR	1900	5° 28.014' N	75° 53.443' W
P. celsiae sp. nov.	MHUA-R13873	paratype	AA_7069	male	Risaralda	Mistrató	MPNR	1900	5° 28.014' N	75° 53.443' W
P. celsiae sp. nov.	MHUA-R13874	paratype	AA_7070	male	Risaralda	Mistrató	MPNR	1900	5° 28.014' N	75° 53.443' W
P. celsiae sp. nov.	MHUA-R13875	paratype	AA_7071	male	Risaralda	Mistrató	MPNR	1900	5° 28.014' N	75° 53.443' W
P. celsiae sp. nov.	MHUA-R13876	paratype	AA_7072	male	Risaralda	Mistrató	MPNR	1900	5° 28.014' N	75° 53.443' W
P. celsiae sp. nov.	MHUA-R13877	paratype	AA_7073	male	Risaralda	Mistrató	MPNR	1900	5° 28.014' N	75° 53.443' W
P. celsiae sp. nov.	MHUA-R13878	paratype	AA_7074	female	Risaralda	Mistrató	MPNR	1900	5° 28.014' N	75° 53.443' W
P. celsiae sp. nov.	MHUA-R13879	paratype	AA_7161	male	Risaralda	Mistrató	MPNR	1900	5° 28.014' N	75° 53.443' W
P. celsiae sp. nov.	MHUA-R13880	paratype	AA_7172	male	Risaralda	Mistrató	MPNR	1900	5° 28.014' N	75° 53.443' W
P. odinsae sp. nov.	MHUA-R13907	**holotype**	AA_7090	male	Antioquia	Jardín	MPNR	2180	5° 29.759' N	75° 53.346' W
P. odinsae sp. nov.	MHUA_R12574	paratype		male	Antioquia	Andes	Santa Rita	2150	5° 35.520' N	75° 57.154' W
P. odinsae sp. nov.	MHUA-R12584	paratype		juvenile	Antioquia	Jericó	Quebradona	2240	5° 45.378' N	75° 43.365' W
P. odinsae sp. nov.	MHUA-R12986	paratype		juvenile	Chocó	El Carmen*	La Isla	1730	5° 51.502' N	76° 9.727' W
P. odinsae sp. nov.	MHUA-R13883	paratype	AA_7009	male	Antioquia	Jardín	MPNR	1920	5° 31.619' N	75° 51.754' W
P. odinsae sp. nov.	MHUA-R13884	paratype	AA_7011	female	Antioquia	Jardín	MPNR	1920	5° 31.619' N	75° 51.754' W
P. odinsae sp. nov.	MHUA-R13885	paratype	AA_7012	female	Antioquia	Jardín	MPNR	1920	5° 31.619' N	75° 51.754' W
P. odinsae sp. nov.	MHUA-R13886	paratype	AA_7013	male	Antioquia	Jardín	MPNR	2300	5° 31.127' N	75° 51.737' W
P. odinsae sp. nov.	MHUA-R13887	paratype	AA_7015	male	Antioquia	Jardín	MPNR	1920	5° 31.619' N	75° 51.754' W
P. odinsae sp. nov.	MHUA-R13888	paratype	AA_7016	female	Antioquia	Jardín	MPNR	2210	5° 29.615' N	75° 53.401' W
P. odinsae sp. nov.	MHUA-R13889	paratype	AA_7019	juvenile	Antioquia	Jardín	MPNR	2300	5° 31.127' N	75° 51.737' W
P. odinsae sp. nov.	MHUA-R13898	paratype	AA_7182	female	Antioquia	Jardín	MPNR	2310	5° 29.457' N	75° 53.327' W
P. odinsae sp. nov.	MHUA-R13899	paratype	AA_7183	female	Antioquia	Jardín	MPNR	2310	5° 29.457' N	75° 53.327' W
P. odinsae sp. nov.	MHUA-R13904	paratype	AA_7188	male	Antioquia	Jardín	MPNR	2230	5° 30.962' N	75° 50.629' W
